# The possible role of oxidative stress marker glutathione in the assessment of cognitive impairment in multiple sclerosis

**DOI:** 10.1515/med-2024-0952

**Published:** 2024-04-10

**Authors:** Andrijana Bogoje Raspopović, Vedran Balta, Maro Vodopić, Marina Drobac, Almoš Boroš, Domagoj Đikić, Vida Demarin

**Affiliations:** Department of Neurology, General Hospital Dubrovnik, Dubrovnik, Croatia; Department of Animal Physiology, Biology Division, Faculty of Science, University of Zagreb, 10000 Zagreb, Croatia; Czech Academy of Science, Institute of Physiology, Prague, Czechia; Croatian Academy of Sciences and Arts, Zagreb, Croatia; Department of Animal Physiology, Biology Division, Faculty of Science, University of Zagreb, Rooseveltov trg 6, 10000 Zagreb, Croatia

**Keywords:** glutathione, oxidative stress, multiple sclerosis, MoCA test

## Abstract

Oxidative stress markers have a distinct role in the process of demyelination in multiple sclerosis. This study investigated the potential correlation of markers of oxidative stress (glutathione [GSH], catalase) with the number of demyelinating lesions and the degree of disability, cognitive deficit, and depression in patients with relapsing-remitting multiple sclerosis (RRMS). Sixty subjects meeting the criteria for RRMS (19 men and 41 women), and 66 healthy controls (24 men, 42 women) were included. In this study, GSH significantly negatively correlated with the degree of cognitive impairment. This is the first study of subjects with RRMS that performed the mentioned research of serum GSH levels on the degree of cognitive damage examined by the Montreal Scale of Cognitive Assessment (MoCA) test. The development of cognitive changes, verified by the MoCA test, was statistically significantly influenced by the positive number of magnetic resonance lesions, degree of depression, expanded disability status scale (EDSS), age, and GSH values. Based on these results, it can be concluded that it is necessary to monitor cognitive status early in RRMS patients, especially in those with a larger number of demyelinating lesions and a higher EDSS level and in older subjects. Also, the serum level of GSH is a potential biomarker of disease progression, which could be used more widely in RRMS.

## 1 Introduction

Multiple sclerosis (MS) is a chronic inflammatory demyelinating disease of the central nervous system (CNS) with an immune and neurodegenerative component. The pathophysiological mechanisms involved in the onset and progression of MS include inflammation, demyelination, axonal and neuronal damage, oxidative stress, and excitotoxicity [1]. Oxidative stress participates in the inflammatory and neurodegenerative phase of MS. It is a condition caused by an imbalance between the formation of oxidative metabolites and their elimination by the cellular antioxidative defense system that includes an array of enzymes such as superoxide dismutase, catalase, and the system of reduced glutathione (GSH) and its accompanying enzymes GSH reductase and peroxidase. The oxidative state and redox imbalance in the cells are caused by reactive oxygen species (ROS) that are produced by oxidative stressors originating from xenobiotics, pathophysiological conditions, and partial also produced by the normal mitochondrial metabolism, of oxidizing enzymes and essential cellular constituents [2]. ROS are highly reactive molecules that play important roles in various physiological cellular processes, such as cell signaling, gene expression, and host defines; however, increased levels of ROS induce oxidative stress, which is a common pathological feature of neurological disorders, including MS [3].

Cells of the brain and nervous system are prone to oxidative damage due to the relatively low content of antioxidative defense system, especially enzymes, and because of their high content of membrane polyunsaturated fatty acids and iron that are easily released from damaged cells. To protect against ROS-induced damage and cell death, cells are equipped with elaborate antioxidant mechanisms and under physiological conditions, cellular ROS levels are in balance with this endogenous antioxidant system. However, when this balance is altered by increasing ROS production or decreasing the expression or activity of antioxidative enzymes and GSH, there is an increase in oxidative stress and subsequent damage to proteins, lipids, cellular proteins, and DNA of neurons [4]. Overall, this suggests that detoxification and antioxidant protection against ROS are important processes within the brain and many papers have highlighted the importance of oxidative stress in the pathogenesis of MS [5]. In the initial phase of MS lesion formation, locally produced ROS can cause disruption of the blood–brain barrier (BBB) and promote leukocyte migration. *In vitro* studies show that ROS promote BBB permeability, causing cytoskeletal rearrangements in endothelial cells, and redistribution and loss of tight junctions [6]. These monocyte-induced barrier changes and subsequent monocyte migration can be prevented by ROS scavengers, proving that ROS play a key role in leukocyte transendothelial migration. ROS produced by infiltrating leukocytes and resident microglia are thought to contribute to MS pathology, promoting myelin phagocytosis and causing oligodendrocyte cell death induced by oxidative damage, axonal injury, and mitochondrial dysfunction [3]. Oxidative stress is a trigger for the activation of antioxidant mechanisms in cells.

GSH is the major antioxidant in the brain and as such plays a pivotal role in the detoxification of reactive oxidants [7,8]. GSH homeostasis is altered in MS and has the potential of an *in vivo* biomarker in MS, as it enables assessment of the oxidation state in patients with MS and monitoring of disease progression. In the cells of the CNS, the concentration of GSH is about 1–2 mmol/L, and it is several hundred times higher in the cerebrospinal fluid, which is ∼4 μmol/L, and in the blood, which is ∼2 μmol/L. To maintain this rather different concentration ratio, intracellular GSH synthesis is required [4]. It is thought that although GSH from the blood enters the brain via transporters in brain capillaries and endothelial cells across the BBB, the majority is resynthesized by astrocytes and neurons and is indispensable for several important processes related to antioxidants of both cell types. It acts as a cofactor in detoxification reactions and is involved in non-enzymatic and enzymatic inactivation of free radicals. GSH is also important in the storage and transport of cysteine and the maintenance of protein sulfhydryl groups in a reduced form [8]. Neurons and oligodendrocytes are more sensitive to oxidative stress than astrocytes, which generally have the highest levels of GSH. Moreover, astrocytes have a more efficient GSH metabolism, since they are able to utilize a wider variety of precursor substrates for GSH synthesis. On the other hand, neurons, although capable of synthesizing GSH, depend on neighboring astrocytes for the supply of GSH precursors. Neuronal GSH content is not replenished by direct GSH uptake, because the extraordinary concentration of GSH is very low and would therefore cost a disproportionate amount of energy. Active MS lesions show a high level of expression of glutaminase, which converts glutamine to glutamate in macrophages and microglia in the immediate vicinity of dystrophic axons, indicating that glutamate production by macrophages could underlie axonal degeneration and oligodendrocyte death in MS. Consistent with this, several studies show that oligodendrocytes have particularly low levels of GSH, making them more susceptible to damage caused by oxidative stress [7].

MS is a complex disease, and many clinical and pathological factors are involved in cognitive disorders, including depression and fatigue, but also fluctuating inflammation and activity of the immune process [9]. Cognitive dysfunction in MS affects certain functions more often than others; mainly frontal and temporal structures are affected, which play a major role in emotional functions; therefore, the connection between cognitive and emotional disorders may be related to the area of damage [10]. Brain damage visualized by magnetic resonance (MR) correlates with the results of neurophysiological tests. White matter lesions and cortical atrophy are good predictors of cognitive impairment in MS. The results of numerous MR studies indicate that damage to the white and grey matter of the brain is involved in the development of cognitive disorders [11]. Changes in white matter change connections within neural networks, thereby reducing the speed of thought processing, disrupting attention, and working memory, and changes in grey matter can result in changes in memory and behavior.

The objective of this study was to investigate the potential correlation of markers of oxidative stress (GSH, catalase) in certain phases of relapsing-remitting multiple sclerosis (RRMS), and to additionally correlate them with the number of demyelinating T2/FLAIR lesions.

Furthermore, the goal is to determine whether these changes in the correlation ratios of the mentioned physiological parameters correlate with the degree of disability, cognitive deficit, and depression in patients with RRMS.

## 2 Materials and methods

### 2.1 Study population

The study was conducted in the General Hospital Dubrovnik from September 2020 to October 2021.

The subjects were diagnosed based on the revised McDonald’s criteria with the inclusion of criteria (duration of the disease less than 10 years, clinical confirmation of at least one relapse in a year, expanded disability status scale [EDSS] number less than 5.0). According to the revised McDonald’s criteria from 2017, the diagnosis of MS is based on clinical and paraclinical criteria (MR, cerebrospinal fluid diagnostics) which are based on two basic postulates, dissemination in space and dissemination in time, which can be satisfied clinically and/or magnetic resonance imaging. The criterion of dissemination in space is met when we have clinical or MR signs of damage in at least two systems, and dissemination in time is satisfied if the patient has either clinical or neuroradiological evidence that two damages occurred at different times, or positive IgG oligoclonal bands. There were no relapses within 3 months before cognitive testing.


**Ethical approval**: The study had been approved by the Medical Ethics Committee of General Hospital Dubrovnik and School of Medicine Zagreb (380-59-10106-16-20/157).
**Informed consent**: The patients and their families understood the research content and methods and agreed to sign the corresponding informed consent.

### 2.2 Biological markers of antioxidative defense in serum

Blood samples were collected in the morning, and after 30 min, they were centrifuged for 15 min at 1,000×*g*. The absolute serum concentrations of oxidative stress markers (GSH, catalase) were determined by spectrophotometric reading. Oxidative stress marker samples were stored at −70°C, all according to the kit manufacturer’s instructions.

The determination of catalase (CAT) activity in samples relied on the H_2_O_2_ degradation rate in the reaction mix. The reaction was initiated by adding 100 µL of sample to 900 µL of the reaction mixture containing 33 mmol/L H_2_O_2_ in 50 mmol/L phosphate buffer (pH 7.0). The absorbance at 240 nm (Libro S22 Spectrophotometer Biochrom Ltd. Cambridge, UK) was recorded for 3 min, and CAT activity was calculated from mean absorbance change per minute and molar absorption coefficient for H_2_O_2_ (43.6 mol/L cm). The results are expressed as U/mg protein.

Reduced GSH concentrations were determined following Ellman’s method as described elsewhere [8,12,13]. Briefly, 20 µL of sample was incubated with 40 µL of 35 mmol/L HCL for 10 min. At the same time, we prepared the enzyme working solution by adding 20 µL of GSH reductase (0.2 U/mL) to 9.98 mL of NADPH (0.8 mmol/L). The reaction mixture was prepared in a 96-well plate by pipetting 40 µL of 10 mmol/L DTNB, pretreated sample, and 100 µL of enzyme working solution. The absorbance at 412 nm was monitored for 5 min (ELISA plate reader, BIORAD Laboratories, Hercules CA, USA) to obtain the mean change per minute. GSH concentration was calculated using the calibration curve, and the results are reported as nmol/L per mg of proteins.

### 2.3 Cognitive and parameters of CNS damage

All subjects (with RRMS and the control group) underwent an magnetic resonance imaging (MRI) of the CNS (brain and cervical spinal cord), within 1 week after blood sampling, on a 1.5 Tesla (Siemens Somatom Essenza). Images are taken in T1, T2, and FLAIR measurement times, in sagittal, coronal, and transverse sections and analyzed by neuroradiologist.

EDSS is the gold standard scale for assessing the disability of MS patients. This scale quantitatively assesses the degree of clinical damage for individual systems (pyramidal, sensory, brainstem system, cerebellum, visual system, cognitive functions, others). Based on the number in those systems, the total EDSS number is calculated, which gives neurologists an objective quantification of disability, and the total value can be from 0 to 10, with EDSS values of 0–4.5 referring to patients who are fully ambulatory, while values of 5.0–9.5 are defined with movement difficulties [14].

The Montreal Scale of Cognitive Assessment (MoCA) was designed as a quick test to screen patients with mild cognitive impairment, a function that includes different cognitive domains: attention, concentration, executive functions, memory, language, visual construction abilities, conceptual thinking, calculation, and orientation [15]. The time of application of the MoCA test is approximately 10 min. The total possible score is 30 points, a score of 26 points or more is considered normal.

The Beck Depression Inventory II (BDI-II) is a self-report scale consisting of 21 questions, each of which can be answered with four answers that are graded from 0 to 3. The minimum grade is 0, and the maximum is 63. The questions refer to mood disorder, loss hopes, feelings of rejection, inability to enjoy, feelings of guilt, need for punishment, self-hatred, self-judgment, tendency suicide, tearfulness, irritability, disturbance in relationship with other people, indecisiveness, negative self-image, inability of work, sleep disturbance, fatigue, lack of appetite, weight loss, hypochondria and loss of libido. The average results show that respondents who scored up to 11 points do not have depression, and respondents who scored between 12 and 28 points have mild or moderate depression [16]. A major depressive episode is diagnosed when a score greater than 28 is reached [17].

### 2.4 Statistical analysis

Statistical data processing was carried out using the SPSS statistical program. The quantitative analytical paradigm was applied in the data analysis. The results of the quantitative data analysis were interpreted with at least a 5% level of significance and analyzed with the help of the program support STATISTICS 11. Stat Soft. Inch. The distribution of qualitative data was presented with contingency tables, and the data were analyzed with the *χ* ^2^ test or, if necessary, with Fisher’s exact test. Distributions of quantitative measurements (properties) are tested for normality with the Smirnov-Kolmogorov test and depending on the outcome in the description and analysis, some of the appropriate statistical-analytical procedures were applied (Student’s *T* test, Mann–Whitney *U* test, analysis of variance [ANOVA], Kruskal–Walis ANOVA test). Correlation and regression models are used in data analysis with regard to data distribution (e.g., Spearman’s correlation).

## 3 Results

A total of 60 subjects meeting the criteria for RRMS (19 men and 41 women) and 66 healthy control subjects (24 men, 42 women) were included in the study (Table 1). The respondents are uniform in age (Table 1). The average age of healthy and RRMS patients is around 43 years.

**Table 1:** Profile of respondents by age and sex

		*N*	Mean	SD	Min	Max	Centile		
Group							25.	75.	Sig.(−2 tailed)
Age (years)	Healthy	66	43.50	9.58	20.00	60.00	32.00	48.00	0.300
	RRMS	60	43.50	10.76	18.00	66.00	36.00	50.00	

		Group				Sig.(−2 tailed)
		Healthy		RRMS		
		*N*	%	*N*	%	
Sex	Male	24	36.4	19	31.7	0.579
	Female	42	63.6	41	68.3	

In the group of RRMS patients, there were significantly (*p* < 0.001) lower GSH values and significantly (*p* < 0.001) lower CAT values (Table 2).

**Table 2:** GSH and CAT test results

		*N*	Mean	SD	Min	Max	Centile		Sig.(−2 tailed)
Group							25.	75.	
GSH (µmol/mg proteins)	Healthy	66	14.83	26.56	5.77	227.64	12.04	16.60	<0.001*
	RRMS	60	11.06	2.83	1.74	19.61	9.30	12.79	
CAT (U/mg proteins)	Healthy	66	0.25	0.12	0.07	0.61	0.18	0.35	
	RRMS	60	0.14	0.13	0.03	0.67	0.08	0.20	

*Significant difference (*p* < 0.001) between RRMS and healthy subjects.

The cognitive functions of subjects with the MoCA test were statistically significantly different (<0.001) between subjects with RRMS and the control group, because subjects with RRMS have cognitive deviations and lower values on the MoCA test. The BDI-II test showed higher values in patients with MS compared to the healthy, which indicates the development of depression (Table 3).

**Table 3:** Comparison of respondents according to the MoCA and BDI-II test

		*N*	Arithmetic mean	SD	Min	Max	Centile			Sig.(−2 tailed)
Group							25.	Median	75.	
MoCA	Healthy	66	28.91	1.33	24.00	30.00	28.00	29.00	30.00	<0.001*
	RRMS	60	26.15	2.37	20.00	30.00	25.00	26.00	28.00	
BDI-II	Healthy	66	2.29	2.15	0.00	10.00	0.00	2.00	4.00	
	RRMS	60	10.22	4.95	1.00	27.00	6.00	9.00	14.00	

*Significant difference (*p* < 0.001) between RRMS and healthy subjects.

The oxidative stress marker GSH was statistically significantly negatively correlated with the MoCA test (Table 4).

**Table 4:** Correlations of GSH, MoCA test, and the number of MR lesions; T1, T2, and FLAIR with all investigated parameters

	Correlation coefficient	Sig. (2-tailed)	*N*
**GSH**			
EDSS	0.180174062	0.1683	60
Age (years)	0.032954669	0.8026	60
CAT (U/mg serum proteins)	0.148874687	0.2563	60
MoCA	−0.316597057	0.0137*	60
BDI-II	0.104938039	0.4249	60
MR lesions T1, T2, FLAIR	0.231157334	0.0756	60
**MoCA**			
EDSS	−0.427345919	0.0007*	60
Age (years)	−0.449401242	0.0003*	60
GSH (µmol/mg serum proteins)	−0.316597057	0.0137*	60
CAT (U/mg proteins)	0.016959555	0.8977	60
BDI-II	−0.291691917	0.0237*	60
MR lesions: T1, T2, FLAIR	−0.570659701	0.0000*	60
**MR lesions T1, T2, FLAIR**			
EDSS	0.384182718	0.002441562*	60
Age (years)	0.211634789	0.104530229	60
GSH (µmol/mg serum proteins)	0.231157334	0.075564216	60
CAT (U/mg proteins)	0.097566407	0.458317424	60
MoCA	−0.570659701	1.93063 × 10^−6^*	60
BDI-II	0.204672601	0.116719239	60

*Statistically significant correlation (*p* < 0.005).

In our study, there was no significant correlation between EDSS and GSH level, presumably because the level of GSH was not high enough to protect neurons from oxidative damage. We assume if EDSS was lower, it could possibly correlate with GSH.

The MoCA test for cognitive functions statistically significantly correlated with EDSS, age, BDI-II, and MR lesions: T1, T1, FLAIR, and GSH. Subjects with higher MoCA test values were younger, had a lower degree of neurological disability, were less depressed, and had lower GSH values and fewer demyelinating lesions on MRI.

The number of demyelinating lesions shown by magnetic resonance in T1, T2, and FLAIR sequences showed a statistically significant positive correlation with the degree of neurological disability (EDSS) and the MoCA test. Subjects with a higher number of demyelinating lesions had a higher EDSS and a worse score on a cognitive test (Table 4).

In the multivariate regression model, we tried to predict the GSH level. The regression model explains 5% of the variance of the dependent variable (GSH) and it is not statistically significant.

## 4 Discussion

In this study, subjects with RRMS had, as expected, a higher level of EDSS, more points on the Beck self-rating scale, and a higher number of MR lesions and lower results obtained with the MoCA test.

### 4.1 GSH in RRMS

GSH values are higher in healthy subjects compared to subjects with MS, as published in the study by Lubisavljević et al. [18]. They included 50 patients with clinically isolated syndrome (CIS) and 57 patients with RRMS. Significantly reduced GSH values were obtained in both examined groups in comparison with control values. Depletions were more pronounced in RRMS than in CIS patients (*p* = 0.009 for GSH). The results show that GSH can be important in the development of neuroinflammation and serve as a marker that is closely related to neurological and radiological signs of acute inflammation of the CNS. In the study by Choi et al. [19] lower GSH values were also found in 21 subjects with RRMS, 20 with SPMS, and 20 with PPMS compared to 28 healthy controls. The results have shown markedly lower GSH in progressive MS than in RRMS, indicating a more pronounced involvement of oxidative stress in the progressive stage of MS than in the inflammatory stage. The connection between GSH and brain atrophy indicates an important contributing role of oxidative stress neurodegeneration in progressive MS. Calabrese et al. [13] showed significantly lower GSH values in the cerebrospinal fluid of patients with MS, compared to controls, which probably reflects lower GSH in the CNS. A 2011 study by Van Horssen et al. [3] shows that oligodendrocytes have particularly low levels of GSH, making them more sensitive to oxidative stress, and in this way, they also produce reduced ability to restore the myelin sheath. Krotenko et al. [20] analyzed the peripheral blood of 79 MS patients and 75 healthy patients. They found lower GSH levels in RRMS patients compared to controls. The authors report a lower level of GSH in the phase of remission and in the phase of exacerbation, both in RRMS and secondary progressive MS. Di Giussepe et al. [21] measured GSH in very fluid plasma chromatography and concluded that there are no significant differences in GSH concentration between healthy and MS patients. In the studies of Tasset et al. [22] on 24 patients with RRMS and 15 healthy controls, GSH levels in the blood of patients with MS were increased, which may be partly explained by an increase in the activity of GSH reductase, which regenerates GSH from oxidation form of GSH. Given the variation in blood measurements of GSH and GSH-related enzymes and cerebrospinal fluid in different studies, imaging methods that allow non-invasive quantification of brain GSH are useful in providing accurate measurements in real time. One of the causes of increased GSH was shown by stimulation of extracellular glutamate, indicating that GSH may have neuroprotective role to reduce glutamate toxicity and thus affect neurodegeneration [23]. Also, the reduced level of GSH in the brain, primarily in the white matter, should be taken into account, as it can be used to identify patients who have a higher risk of progression disease, and should be investigated in terms of how this reduction affects functional and cognitive activity [19,23].

### 4.2 GSH and MoCA in RRMS

In this study, GSH was significantly negatively correlated with the degree of cognitive impairment (MoCA test, Table 4), although we did not find the statistically significant correlation in the multivariant model (Table 5). This is the first study of subjects with RRMS that did the aforementioned research. It is likely that changes in regional GSH levels in the brain may affect cognitive and sensorimotor function, although it is unclear whether this relationship was seen in normal aging or only in pathological conditions. In previous research, GSH was shown as a potential marker, therefore we conducted our research. In the similar research of Hupfeld et al. [24], who tested the association between brain GSH levels and cognitive performance in 37 young (mean age 21.8) and 37 elderly (mean age 72.8) healthy individuals, predicted regionally specific relationships in which frontal GSH levels would be related to cognitive performance, and sensorimotor GSH levels were related to motor performance. They concluded that there was no association between GSH levels and cognitive performance determined by the MoCA test, although MoCA test results are sensitive enough to identify associations between neurometabolites measured by magnetic resonance spectroscopy and cognitive status. Based on this research, it could be said that correlations between GSH and cognitive deficit appear only in cases of more serious cognitive decline, when brain resources (such as the availability of antioxidants) have significantly decreased, that is, in pathological conditions of the CNS. The negative correlation of GSH levels and MoCA test results in this study could indicate a compensatory and neuroprotective response of GSH to oxidative stress and associated tissue damage in the brain [24], suggesting a GSH response to increased oxidative stress in RRMS. The limitation of the study is a small number of patients. Future investigations should be conducted on a larger number of patients with possible distinctions with all MS types.

**Table 5:** Multivariate prediction of GSH level

	Unstandardized coefficients		Standardized coefficients	*t*	95.0% CI		*P*
	*B*	Std. error	Beta		Lower bound	Upper bound	
Age (years)	−0.02	0.04	−0.095	−0.67	−0.100	0.050	0.506
EDSS	0.29	0.41	0.115	0.71	−0.534	1.120	0.481
MoCA	−0.396	0.202	−0.371	−1.959	−0.807	0.015	0.058
BDI-II	−0.01	0.08	−0.026	−0.17	−0.184	0.155	0.864
MR lesions: T1,T2, FLAIR	0.01	0.01	0.144	0.93	−0.016	0.043	0.355

## 5 Conclusions

In this study, a statistically significant influence of serum oxidative stress marker GSH on the presence of cognitive changes in subjects was demonstrated. It significantly negatively correlated with the degree of cognitive impairment (MoCA test). This is the first study of subjects with RRMS that performed the mentioned research of serum GSH levels on the degree of cognitive damage examined by the MoCA test. Regardless of the limitations of the study, we can conclude that these results indicate that GSH has the potential to be included in future scientific research as a potential biomarker with cognitive tests in MS.
